# Root growth, function and rhizosphere microbiome analyses show local rather than systemic effects in apple plant response to replant disease soil

**DOI:** 10.1371/journal.pone.0204922

**Published:** 2018-10-08

**Authors:** Maik Lucas, Alicia Balbín-Suárez, Kornelia Smalla, Doris Vetterlein

**Affiliations:** 1 Department of Soil System Science, Helmholtz Centre for Environmental Research–UFZ, Halle/Saale, Germany; 2 Soil Science, Martin-Luther-University Halle-Wittenberg, Von-Seckendorff-Platz 3, Halle/Saale, Germany; 3 Julius Kühn-Institut, Federal Research Centre for Cultivated Plants (JKI), Institute for Epidemiology and Pathogen Diagnostics, Braunschweig, Germany; Estacion Experimental del Zaidin - CSIC, SPAIN

## Abstract

Apple replant disease (ARD) is the phenomenon of soil decline occurring after repeated planting of apple trees at the same site. This study aimed to elucidate whether ARD is systemic, i.e. whether the contact of parts of the root system with ARD soil causes the whole plant to show poor shoot and root growth. A split-root experiment was conducted with seedlings of ‘M26’, offering the same plant for its root system the choice between the substrates ARD soil (+ARD), γ-sterilized ARD soil (-ARD) or soil from a grass parcel (Control) with the following combinations: +ARD/+ARD, -ARD/-ARD; +ARD/-ARD; +ARD/Control. Root growth was analysed throughout the 34-day growing period. Samples from bulk, rhizosphere and rhizoplane soil were collected separately for each compartment, and analysed by fingerprints of 16S rRNA gene or ITS fragments amplified from total community (TC) DNA. The response of the plant to +ARD was not systemic as root growth in -ARD compartment was always superior to root growth in +ARD soil. Crosswise ^15^N-labelling of the N-fertilizer applied to the split-root compartments showed that nitrate-N uptake efficiency was higher for roots in -ARD soil compared to those in +ARD. Bacterial and fungal community composition in the rhizoplane and rhizosphere of the same plants differed significantly between the compartments containing +ARD/-ARD or +ARD/Control. The strongest differences between the bacterial fingerprints were observed in the rhizoplane and rhizosphere. Bacterial genera with increased abundance in response to ARD were mainly *Streptomyces* but also *Sphingobium*, *Novosphingobium*, *Rhizobium*, *Lysobacter* and *Variovorax*. The strongest differences between the fungal fingerprints were observed in bulk soil. Our data showed that the response of the apple plant to ARD soil is local and not systemic.

## Introduction

### ARD in general

The phenomenon of apple replant disease (ARD), i.e. the reduction of crop productivity in field systems repeatedly planted with apple trees, has been recently reviewed by Mazzola & Manici [1]. It is common to all major apple growing areas of the world, however, in contrast to other replant diseases, the causal agents are still not known. The role of toxins has been discussed intensively, in particular as apple roots contain large amounts of specific polyphenols like phloridzin [2–5]. However, replant disease is persistent in soils for many years, while polyphenols are degraded within months [6]. Treating soil with fumigants or sterilizing it alleviates the problem, while diluting pasteurized soil with only 10% ARD soil is sufficient to reduce apple tree growth. Hence, Mazzola & Manici [1] concluded that biotic components should be the causal agents. *Cylindrocarpon* spp., *Rhizoctonia solani* (Kühn), *Pythium*, *Phytophthora*, and nematodes (*Pratylenchus* spp.) are assumed to contribute to the disease [4].

### ARD affects root growth and function

Replant disease symptoms, consistently observed shortly after planting, do not only occur above ground. Roots were reported to show discolouration, necrotic tips, a smaller number of root hairs, and reduced growth [1,2,4,7,8]. In particular, there is some indication that fine roots (fibrous roots) are affected [4,9], while concentration of the flavonoid phloridzin was highest in older (higher order) roots [4]. Little information is available on the functionality of roots showing ARD symptoms, i.e. whether they are still active in nutrient and water uptake. Alterations could be induced by phloridzin upon decompartmentalization and oxidation, resulting in toxic metabolites disturbing membrane integrity [10].

### ARD caused by biotic factors

Since the 1980s, several studies were conducted on the biological components of ARD [11]. Molecular fingerprinting methods revealed large differences in the composition of bacterial, fungal, and nematode communities in ARD soils compared to healthy ones [8,12,13].

Nematodes such as *Pratylenchus penetrans* were initially suspected to cause ARD [14]. However, in later studies, the influence of *Pratylenchus* and other nematodes was found to be low or negligible [15,16].

*Fungi such as Rhizoctonia solani*, *Phytophthora* spp., *Phythium* spp, *Cylindrocarpon* spp., and *Fusarium* spp. are actually referred to as the most important agents associated with ARD [15,17–21]. In experiments with ARD soils from several countries of Europe, however, no negative link was observed between plants growth and abundance of *Fusarium* and *Rhizoctonia* spp., indicating that *Cylindrocarpon* and *Pythium* were the main cause of ARD in Central Europe [11].

However, even though these microorganisms are often mentioned as inducing symptoms of ARD, many uncertainties remain. About their actual link with ARD usually little or nothing is known [1]. In addition, all the studies on apple trees from different regions of the world revealed that dominant pathogens strongly depended on the site of apple plantation [15]. One of the reasons for this might be that the interplay between the soil microbiome and plants largely depends on environmental conditions (soil type, cropping history, weather) and the physiological state of the plants [11,22].

In addition, contrasting results shown by different studies might be in part related to the methodology of sampling. If root rhizodeposits like phloridzin are the reason for the shift in microbial community, this shift most likely occurs next to the root. Therefore, it is inevitable investigate in detail the apple plant rhizosphere and rhizoplane to better understand the etiology of ARD.

### Is the response to ARD systemic?

Split-root experiments are well-established tools to distinguish between local effects such as the increase in root branching as a response to local nitrate or ammonium placement [23], and systemic effects, i.e. the induction of stomatal closure in response to the root system sensing drought [24], or the systemic suppression of root colonization by rhizobium or mycorrhiza due to Nod factors [25]. Presently it is not known whether the apple plant response to ARD soil is local or systemic. In particular, there is a need to investigate whether (i) the symptoms spread throughout the root system, (ii) the potential causing agents spread throughout the root system and associated rhizoplane, rhizosphere and bulk soil, (iii) the exposure of part of the root system to ARD has an negative impact on shoot growth or whether (iv) a compensation by root growth in the ARD unaffected substrate is possible. A clarification of these points could help to identify the causal agents and mechanisms behind ARD. At the same time the split-root approach is a tool to overcome uncertainty introduced by plant to plant variation in shoot and root growth, as the same individual is exposed to +ARD and control or–ARD soil, respectively. To our knowledge no previous experiments using the split-root approach to investigate ARD have been conducted. However, recently, split-root approach has been successfully applied to investigate the genetic events taking place during the tripartite interaction of *Verticillium dahliae-olive-Pseudomonas fluorescens* PICF7 in olive tree roots [26].

### Objective and experimental approach

The goal of the present study was primarily to unravel whether the response of apple to ARD soil is systemic. Roots of the same plant were grown partly in +ARD soil and partly in γ-irradiated ARD soil (-ARD) or the same soil with no previous apple cropping (Control). In addition, plants were also grown in split-root boxes with both compartments either filled with +ARD soil or -ARD soil. Along with the question of whether the response of apple to ARD is systemic, the objective was to check whether differences in the microbiome composition of the same plant were dependent on the substrates and the different microhabitats (bulk and rhizosphere soil or rhizoplane). We focused on root growth and activity as symptoms of ARD. Root growth was investigated *in situ* by scanning the split-root boxes. Root activity was investigated by crosswise labelling of +ARD or -ARD soil by fertilization with K^15^NO_3_. The microbial communities associated with the bulk and rhizosphere soil and the rhizoplane were investigated by denaturing gradient gel electrophoresis (DGGE) fingerprints of 16S rRNA gene and ITS fragments amplified from total community (TC) DNA. Differentiating bands of the bacterial DGGE fingerprints were characterized by sequencing.

## Materials and methods

### Substrates

Topsoil (0–20 cm) was collected from the experimental field site Ellerhoop, Germany (53° 42’ 51.71” N, 9° 46’ 12.16” E), in March 2016. The field experiment had been established in 2010 with ARD-plots (n = 4) replanted with apple rootstock plants (Bittenfelder) every two years, and grass-plots (n = 4) continuously mown throughout the growing season.

Soil from this field site is a loamy sand classified as Endostagnic Luvisol according to WRB [27].

The field moist soils from ARD-plots and grass-plots were sieved to 2 mm and packed in 15 L autoclaveable bags. Half of the soil from ARD plots was subsequently treated by γ-radiation (>10 kGray, Synergyhealth, Radeberg; compare McNamara *et al*. [28], further denoted ‘-ARD’ soil in contrast to the soil from ARD plots not receiving γ-radiation, denoted ‘+ARD’ soil. Soil from grass plots present at the same site and never planted with apple trees was denoted ‘Control’.

All three substrates received a basal fertilizer dressing consisting of 50 mg N kg^-1^, 25 mg P kg^-1^, 140 mg K kg^-1^, 10 mg Mg kg^-1^. Nutrients were applied as KNO_3_, CaHPO_4_, and MgSO_4_. The nitrogen fertilizer was either applied as standard KNO_3_, with natural abundance of ^15^N or as K^15^NO_3_, consisting of 98 at %^15^N.

### Split-root boxes and experimental design

Split-root boxes consisting of two adjacent compartments (32.5 x 10 x 2 cm, h x w x d) and a transparent front plate were used. The back of the boxes was lined with an irrigation mat and a 30 μm nylon mesh to enable water supply to the soil compartments from the back along the height of the boxes, without providing roots direct access to the irrigation mat. Each compartment was filled with 600 g of the respective substrate (bulk density 1.1 g cm^-3^), and irrigated to 20 vol.%. ‘M26’ plants (see below) were carefully transplanted to the split-root boxes after removing adhering substrate from the pre-culture. Existing root system was divided between the two compartments. The top was covered with a layer of vermiculite to avoid drying of the hypocotyl, and a layer of coarse gravel to reduce evaporation from the surface.

The experiment consisted of four main treatments, varying in the combination of substrates in the two compartments of the split-root boxes: -ARD in both compartments (-ARD/-ARD); +ARD in both compartments (+ARD/+ARD); -ARD in one and +ARD in the other compartment (-ARD/+ARD); one compartment with Control and one with +ARD (Control/+ARD).

For crosswise labelling of soil compartments with ^15^N, two sub-treatments of treatment (-ARD/+ARD) were established (^15^N-ARD/^14^N+ARD), (^14^N-ARD/^15^N+ARD), i.e. the ^15^N labelled nitrogen fertilizer was applied once to the -ARD soil, once to the +ARD soil. The second compartment, like all other treatments, received the same amount of unlabelled nitrogen fertilizer (see above).

### Plant material and growth conditions

Acclimatized *in vitro* apple rootstock ‘M26’ plants, 60 days old, were selected for equal size and distributed evenly among the different treatments, resulting in four biological replicates.

Plants were grown in split-root boxes in a climate chamber for 34 days with a 16 h photoperiod (350 μM m^-2^ s^-1^ PAR). Day and night temperatures were 20 and 18°C, respectively, while relative humidity (70%) was kept constant.

Plants were watered to 20 vol.% every two to three days. Towards the harvest water content was reduced to 18 vol.% to enable a standardized collection of rhizosphere soil.

### Scanning root growth in situ

The split-root boxes were placed into the climate chamber at an angle of about 30°. With this angle most roots were growing towards the transparent front plate where root growth could be observed. Therefore, the surface of the split-root boxes was scanned every two to four days during the 34-day growth period using a photo scanner (EPSON Perfection V700 Photo). The corresponding resolution was 600 dpi, the colour depth was 32 bit. The images were analysed for their root length and root diameter classes (see 2.6).

#### 2.5 Sampling of bulk and rhizosphere soil, rhizoplane and roots

After opening the split-root boxes the stem of the apple plantlet was cut with a sterile scalpel. In each compartment the complete root system was separated from the bulk soil. A toothbrush was used to remove soil adhering to the roots after shaking off loosely attached soil. This soil fraction was termed rhizosphere. To obtain the rhizoplane fraction, the complete root system from each compartment was placed into a falcon tube with 30 mL distilled water and vigorously shaken for 30 seconds. The obtained suspension was centrifuged (10,000 *g* for 30 min at 4ºC). The pellet was re-suspended and transferred to a 2 mL reaction tube and centrifuged again (14.000 *g* for 20 min at 4ºC). The pellets obtained (rhizoplane fraction) were transferred to a lysis tube. 0.5 g of bulk and rhizosphere soil were placed in lysis tubes provided by MP Biomedicals (Santa Ana, CA, USA). The tubes were stored in a freezer at -20°C until DNA extraction. Aliquots of rhizosphere and bulk soil were oven-dried at 65°C for determination of soil dry weights and for C, N, ^15^N analyses.

### Root length and root diameter classes

Root length and diameter classes were measured with WinRHIZO (2009, Regent Instruments Canada Inc.). A colour analysis was done for the images of the split-root boxes. The basis of this analysis is a pixel classification depending on colour classes. These classes differentiate roots from soil. To obtain comparable results, they were defined once and then used for all images.

To get information about the “hidden” part of the root system, root length and diameter classes were also measured destructively after final harvest. For this, half of the washed root system was scanned with 600 dpi and 8 bit and also analysed with WinRHIZO. Between harvest and analysis roots were stored in Rotisol (Roth GmbH, Karlsruhe, Germany). The second half of the roots was dried in an oven for C, N analyses and for calculating the total root dry mass. The latter and the WinRHIZO results were used to calculate total root length of one compartment.

### C, N and ^15^N analyses of plant and soil samples

After 24 h in an oven at 65°C soil samples (rhizosphere and bulk soil) and plant samples (stem and leaves and half of the root system) were ground. C, N and ^15^N analyses were conducted using a coupled system of elementar analyser and quadrupole mass spectrometer (Vario EL cube, Elementar Hanau, Germany; Quadropole MS ESD 100, ICI Bremen, Germany).

### Determination of microbial abundance and diversity

#### TC-DNA extraction and purification

TC-DNA was extracted, from 0.5 g of rhizosphere and bulk soil or the microbial pellet obtained from the roots (rhizoplane) using FastDNA SPIN Kit for soil after a harsh cell lysis step with the FastPrep instrument (MP Biomedicals, Santa Ana, CA, USA) according to the manufacturer’s protocol. The TC-DNA was purified by GENECLEAN SPIN Kit (Qbiogene, Inc., Carlsbad, CA, USA) following the manufacturer’s instructions. The DNA yield was checked on an agarose gel and stored at -20°C.

#### Amplification of bacterial 16S rRNA gene and ITS fragments from TC-DNA

Copy numbers of 16S rRNA gene and ITS were determined by real time quantitative PCR 5’-nuclease assay (qPCR) in a CFX96 Real-Time System (Biorad, Germany) with primer and TaqMan probe as previously described by Suzuki *et al*. [29] and Gschwendtner *et al*. [30], respectively. Amplification conditions, reagents concentrations and standards used in both qPCR were as previously described by Vogel *et al*. [31]. Primer sets and Taqman probes are provided in S1 Table.

All PCRs were performed with purified and 1:10 diluted TC-DNA. To study the bacterial community composition the amplification of the bacterial 16S rRNA gene fragments from TC-DNA was performed with the primers F984GC/R1378 according to Gomes *et al*. [32], except that 0.2 μM primer concentration and 0.025 U μL^-1^ Go Taq polymerase (Promega GmbH, Mannheim, Germany) were used for amplification (25 μL final volume). Group-specific PCRs were performed as described by Weinert *et al*. [33] with primers that allow the amplification of 16S rRNA gene fragments specific for: *Alphaproteobacteria*, *Betaproteobacteria*, *Actinobacteria*, *Pseudomonas*, *Bacillus* and *Streptomyces*, except that for *Bacillus* the reverse universal primer R1494 was used.

To get information about fungal communities, ITS fragments were amplified in a nested PCR with primers ITS1F/ITS4 and ITS1F-GC/ITS2 according to protocols described by Weinert *et al*. [33]. Primer sets with respective references are listed in S1 Table.

#### DGGE analyses

Microbial community composition was studied by Denaturant Gradient Gel Electrophoresis (DGGE) of the amplified 16S rRNA gene or ITS fragments. The analyses were performed in an Ingeny PhorU^2^ system (Ingeny, Goes, The Netherlands) according to Weinert *et al*. [33], and gels were silver-stained as described in Heuer *et al*. [34].

#### Cloning of dominant DGGE bands and sequencing

DGGE bands with higher relative abundance in +ARD treatments comparison to–ARD and control soils were excised from DGGE gels. Between 4–8 bands from the replicates of the same treatment and with the same electrophoretic mobility were pooled together and DNA fragments were extracted as described by Babin *et al*. [35], except that the gel slices were smashed with the help of a sterile pipette tip. One microlitre of the extracted DNA was re-amplified using primer pair F984GC/R1378 (see above) and a DGGE was performed to confirm the electrophoretic mobility of the amplicons. A-Tailing (using GoTaq polymerase), ligation into pGEM-T vector and transformation into *E*. *coli* JM109 (Promega, Madison, VI, USA) was performed as described by Babin *et al*. [35]. Twenty positive clones per band were selected and screened as explained by Smalla *et al*. [36]. Plasmids with the correct insert were extracted with GeneJET Plasmid Miniprep Kit (Thermo Scientific ^TM^) as recommended by the manufactures. Plasmid inserts were sequenced in both directions with standard primers SP6 and T7prom (Macrogen, Amsterdam, Netherlands). Closest relative identification was carried out with the BLASTN search tool using the Reference RNA gene sequences database of the National Center for Biotechnology Information (NCBI, USA).

### Statistics

Differences between means for either side of the split-root boxes were tested by t-test following tests for normal distribution and equal variance. For comparison between the four treatments (-ARD/-ARD; +ARD/+ARD; -ARD/+ARD; Control/+ARD) one factorial ANOVA followed by Tukey’s test for pairwise comparison was conducted.

^15^N isotope abundance was investigated by crosswise labelling the compartments of the treatment -ARD/+ARD with ^15^N nitrogen fertilizer instead of ^14^N nitrogen fertilizer. The four replications established for each labelling treatment (^15^N-ARD/^14^N+ARD; ^14^N-ARD/^15^N+ARD) were analysed separately for ^15^N abundance (n = 4) and were pooled for all other parameters (n = 8).

For the evaluation of root length determination, derived from scanning split-root boxes versus destructive sampling, Pearson correlation coefficient was calculated across all compartments and treatments. For this part of statistics SigmaPlot 11.0 statistics tool was used.

DGGE fingerprints were analysed with the software GelCompar II 6.6., and DGGE profiles were compared pairwise, for each gel, by Pearson correlation indices. As a result, Pearson similarity coefficients were obtained and used for the construction of dendrograms based on the Unweighted Pair Group Method with Arithmetic mean (UPGMA) cluster algorithm. To test for significant differences between the fingerprints of the soil in the compartments (-ARD, +ARD or Control) a permutation test at 10.000 times according to Kropf *et al*. [37] was done with the Pearson similarity coefficients. The test provides dissimilarity values (d-val ue) that indicate the extent of the differences between the DGGE fingerprints of different soil variants. Differences in the qPCR data were revealed with a one factorial ANOVA in conjunction with Tukey’s HSD. For this the software R 3.23 in combination with the package agricolae was used. Prior to statistical analysis bacterial 16S rRNA gene and fungal ITS fragment copy numbers were log-transformed.

## Results

### Root growth

**Fig 1:**
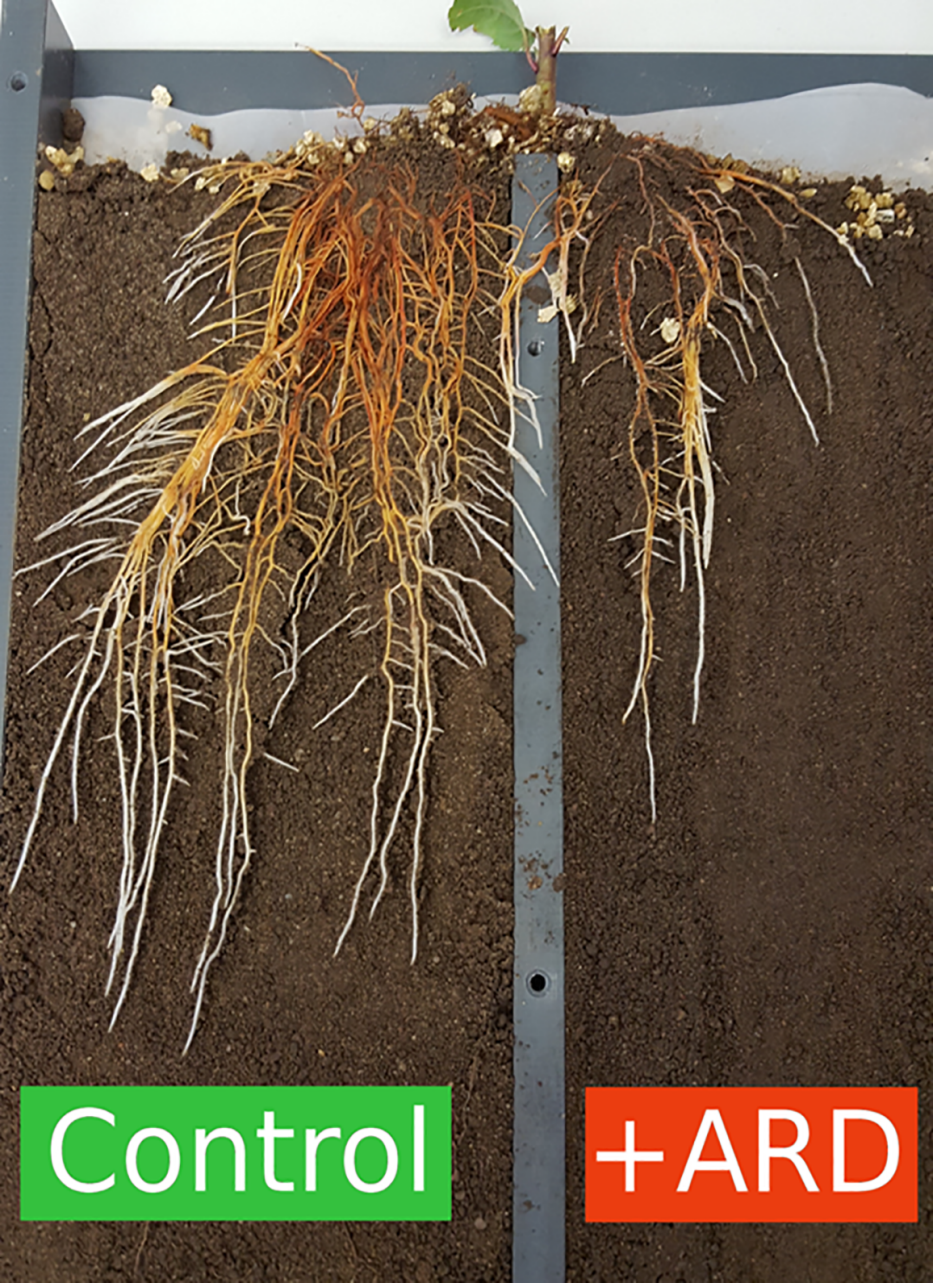
Example of split-root setup showing one representative replicate of treatment Control/+ARD 35 days after planting. Reddish-brown discolouration of roots is visible in both compartments and is typical for apple roots. Colour is related to the high concentrations of phloridzin and quercetin in apple roots.

**Fig 2:**
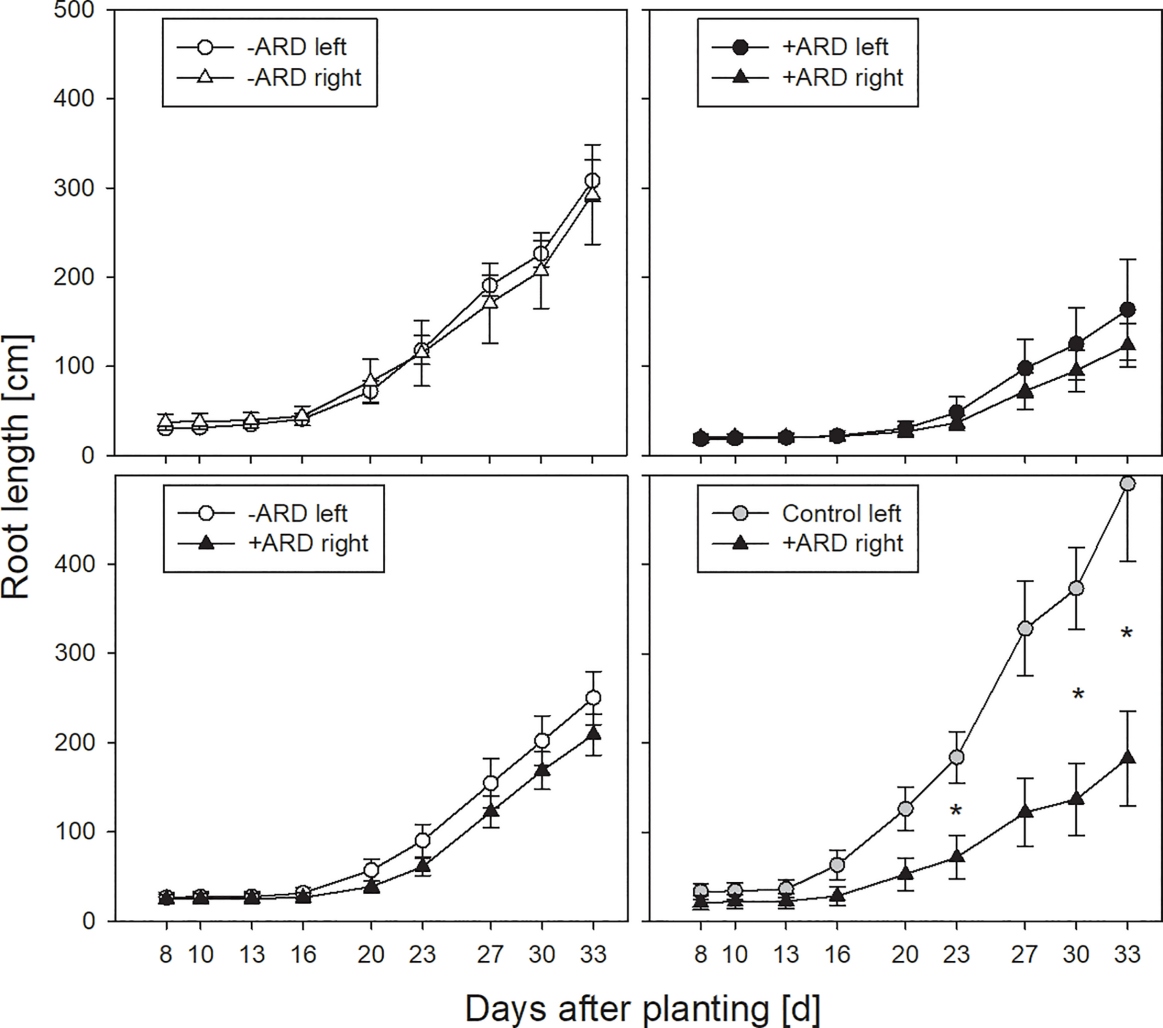
Root length development of apple roots in the two compartments of the split-root boxes for the four treatment combinations: -ARD/-ARD, +ARD/+ARD, -ARD/+ARD, Control/+ARD. Root length development was derived from surface scanning of the split-root boxes at 2–4 days intervals during the 34-day growth period. Asterisks indicate significant differences between the two compartments of one treatment at the respective point in time (p<0.05). Means of four replicates. Error bars show standard errors (SE).

**Fig 3:**
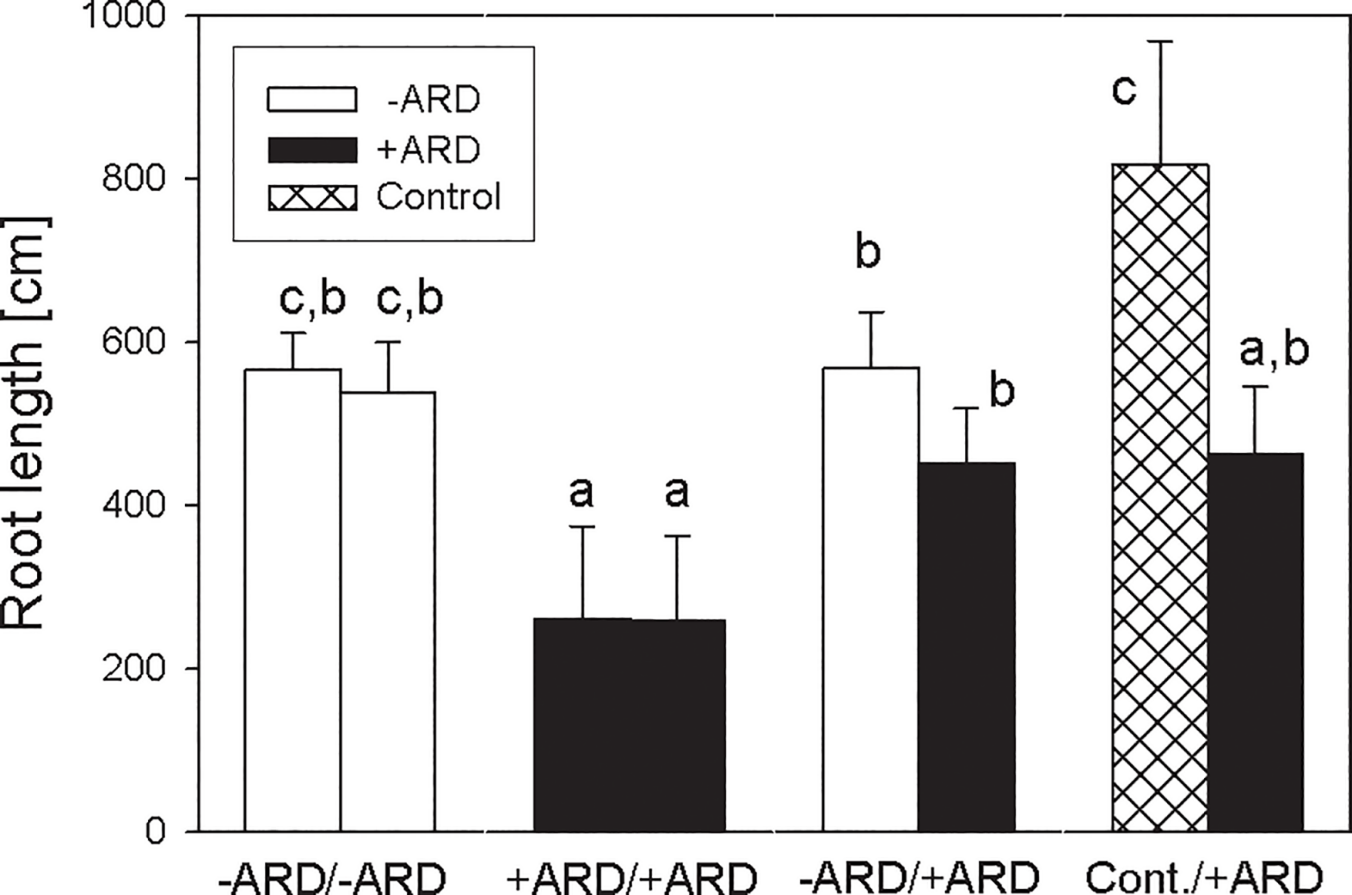
Root length of apple roots in the two compartments of the split-root boxes for the four treatments -ARD/-ARD, +ARD/+ARD, -ARD/+ARD, Control/+ARD 34 days after planting. Root length was determined with WinRhizo after destructive sampling. The substrate in the respective compartment is indicated by colour/pattern code (see legend). Different letters indicate significant differences at p<0.05. Means of four replicates. Error bars show SE.

The split-root box approach appeared well suited for growth of ‘M26’ seedlings, enabling observation of roots throughout the 34-day growth period (Fig 1). The majority of roots was visible through the transparent front plate, and root length data derived from scanning split-root box surface correlated well (r = 0.86*) with the root growth measured destructively at harvest (S1 Fig). After a lag-phase of about 13 days, root length increased and started to differ between treatments and between compartments within one treatment, if these had been filled with different substrates (Fig 2). The most pronounced differences were observed between the two compartments of treatment Control/+ARD, with much higher growth rates in Control compared to +ARD soil. For the treatment -ARD/+ARD there was a tendency for weaker growth in +ARD soil compartment, but differences were not significant. The treatment with +ARD soil in both compartments showed significantly reduced root length compared to the treatments with -ARD in both compartments (Figs 2 and 3). Root length in the +ARD compartment of the treatments with -ARD or Control soil in the second compartment of the split-root box showed intermediate root length (Fig 3).

Differences in root diameter classes among treatments and compartments within each treatment were not observed. More than 80% of the root length showed less than 0.5 mm in diameter, half of which was less than 0.25 mm (data not shown).

### Shoot growth and N uptake

**Fig 4:**
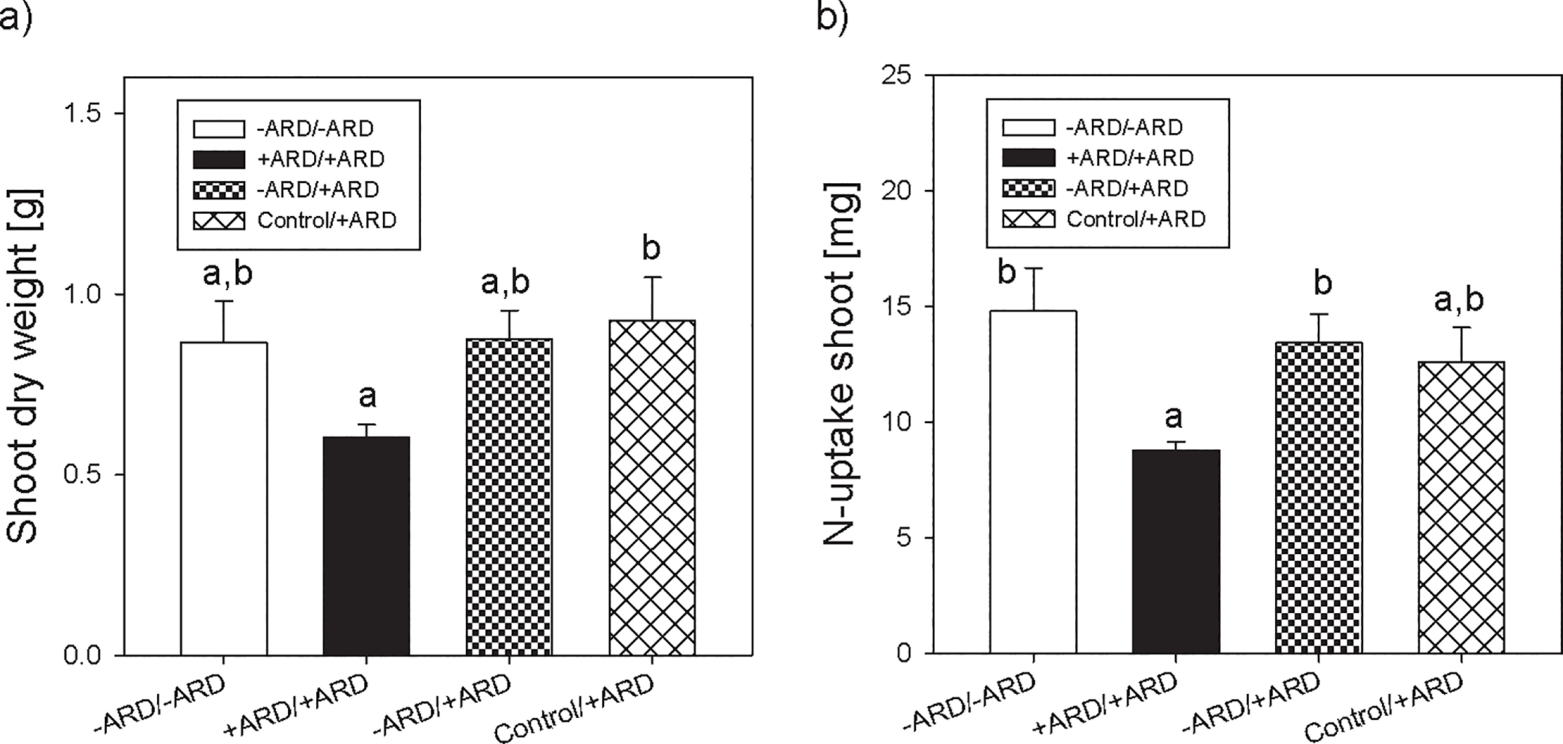
Shoot dry weight (a) and shoot N-uptake (b) 34 days after planting for apple seedling growing on -ARD or +ARD soil only, and treatments with part of their root system growing in +ARD soil vs -ARD soil and control soil, respectively. Different letters indicate significant differences at p<0.05. Means of four replicates. Error bars show SE.

Shoot growth was poor for the treatment +ARD soil only (+ARD/+ARD) compared to -ARD (-ARD/-ARD) (Fig 4A). For treatments -ARD/+ARD and Control/+ARD shoot growth was comparable to the treatment with -ARD/-ARD soil.

Similar results were observed for N uptake into the shoot, i.e. the product of biomass and N concentration in the tissue showing statistically significant differences (Fig 4B).

### ^15^N abundance—Root function

**Fig 5:**
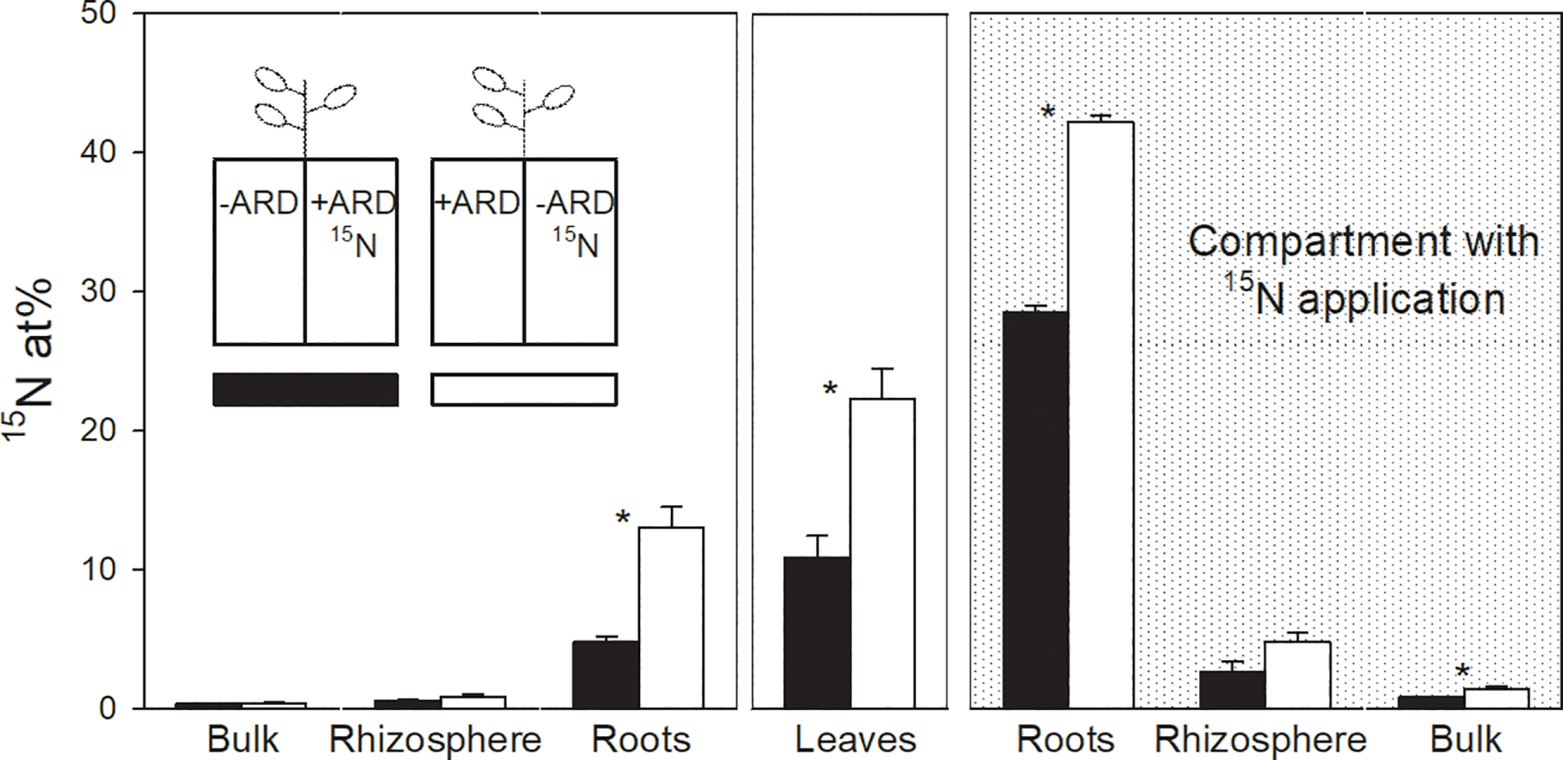
^15^N abundance in different soil and plant fractions after 34-day growing period of apple trees in split-root boxes. Split-root boxes were filled with -ARD soil in one compartment and +ARD soil in the second compartment. Normal nitrogen (^14^N) fertilizer was crosswise replaced with ^15^N labelled nitrogen fertilizer, i.e. in one treatment -ARD soil was labelled with ^15^N (white columns), in the other treatment +ARD soil was labelled with ^15^N (black columns). Grey-shaded background indicates that these samples have been retrieved from the labelled side of the split-root boxes. Asterisk indicates significant differences (p<0.05) between treatments for the respective fraction. Means of four replicates. Error bars show SE.

Crosswise ^15^N labelling was conducted for the treatment -ARD/+ARD to derive information on the activity and functionality of roots grown in +ARD soil compared to those growing in -ARD soil. Higher abundance of ^15^N was observed in the roots and in particular in the shoots if ^15^N was applied to the -ARD soil as compared to +ARD (Fig 5). This was still the case if ^15^N uptake was normalized to root length in the respective compartment (data not shown). Interestingly, some of the ^15^N label was detected in the roots of the unlabelled compartment. ^15^N data indicated that not only uptake activity of roots in -ARD soil was higher but also redistribution of N within the plant was more efficient (Fig 5). Total N concentrations in shoot (1.91%) and root tissue (1.84%) did not differ for the crosswise labelled treatments (data not shown).

### Microbiome analysis

#### Quantification of 16S rRNA gene and ITS fragment copy numbers

**Fig 6:**
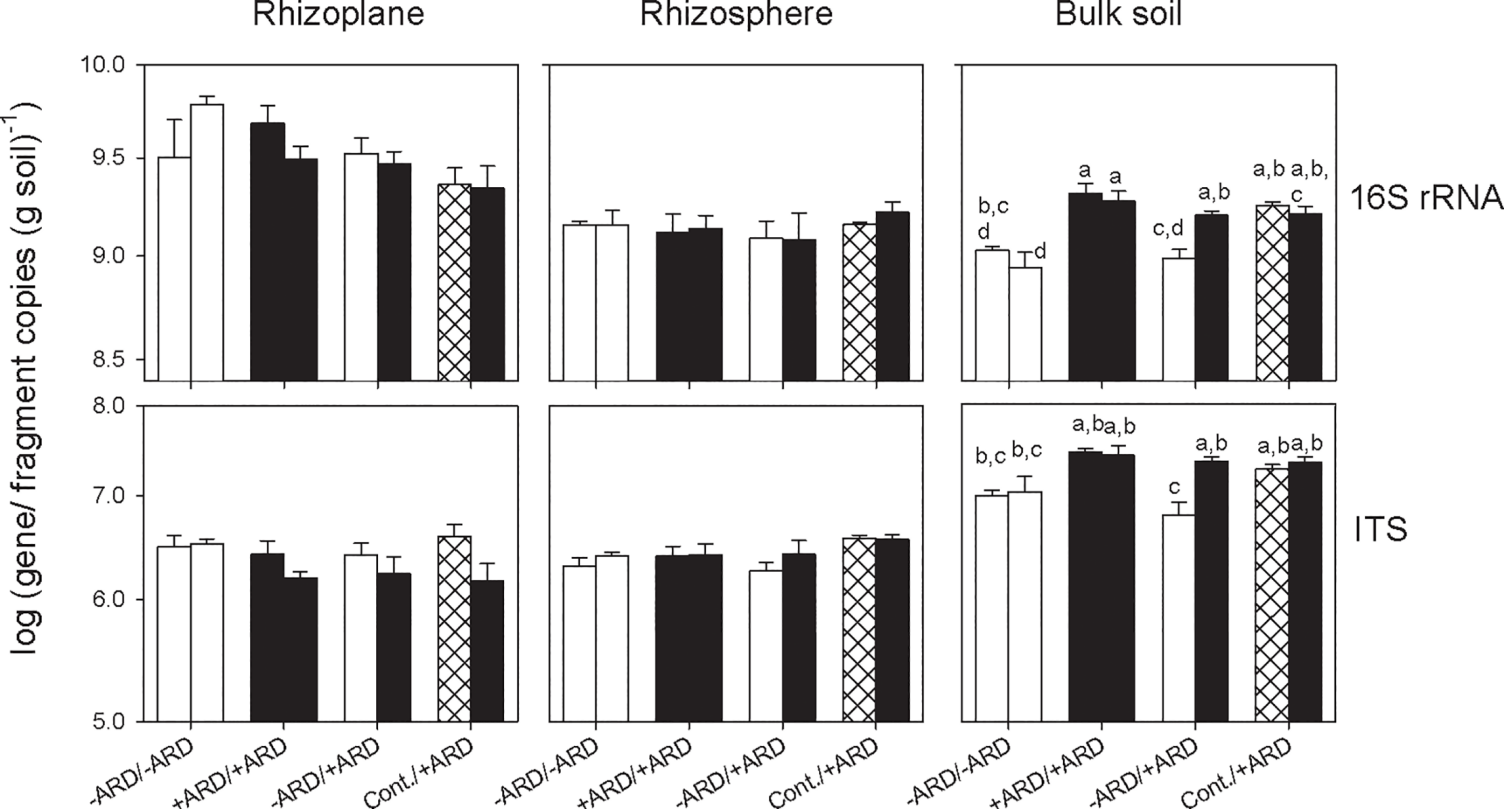
Quantification of 16S rRNA gene and ITS fragment copy numbers in the two compartments of the split-root boxes for the four treatments -ARD/-ARD; +ARD/+ARD; -ARD/+ARD; Control/+ARD based on soil dry weight (bulk and rhizosphere soil) or centrifuged soil sample (rhizoplane), respectively. Data are provided separately for three microhabitats (rhizoplane, rhizosphere and bulk soil) at the end of the 34-day growth period. +ARD soil (black), -ARD soil (white), Control soil (cross-hatched). Different letters indicate significant differences at p<0.05 (only shown for bulk soil). Means of four replicates. Error bars show SE.

Real-time PCR results showed that the 16S rRNA gene copy numbers were higher in the rhizoplane than in rhizosphere and bulk soil for all treatments (Fig 6). In contrast, the ITS fragment copy numbers were higher in the bulk soil than in the rhizosphere and rhizoplane samples. Significant differences between treatments were detected only within the bulk soil samples (Tukey test, n = 4) as significantly lower copy numbers of 16S rRNA gene and ITS fragments were observed in -ARD soil compared to +ARD and Control.

#### DGGE

**Fig 7:**
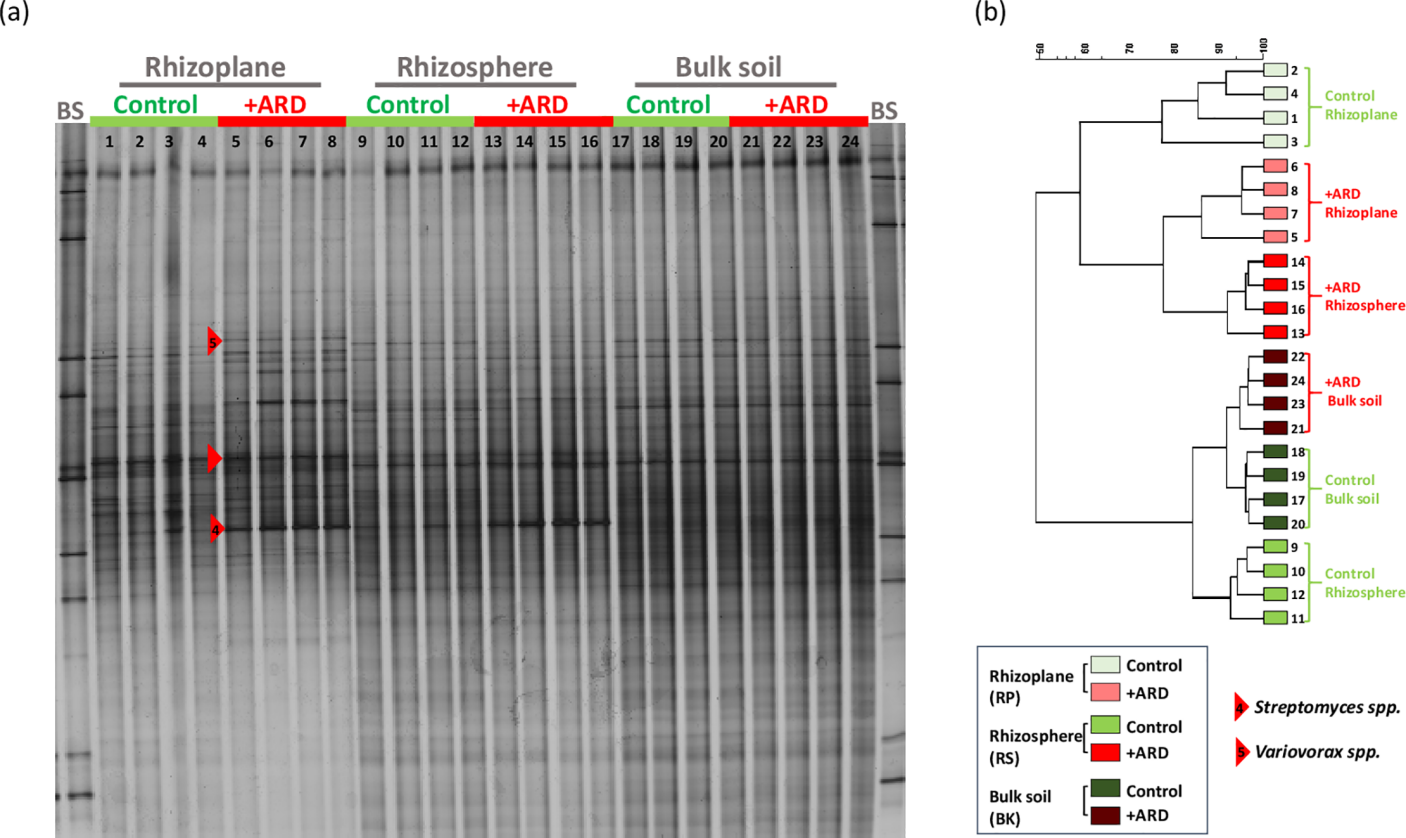
Bacterial response to ARD depending on the microhabitat studied. Bacterial DGGE fingerprints based on 16S rRNA gene fragments amplified from total genomic DNA extracted from different microhabitats (Rhizoplane, Rhizosphere and Bulk soil) from plants grown in Control and +ARD soils, at the same time. On the right side the respective UPGMA cluster analysis of the samples is shown. Red arrows indicate differentiating bands (S3 Fig) detected only in the bacterial fingerprint of ARD soils. The bands were excised, reamplified, cloned and sequenced. BS: Bacterial standard of 16S rRNA gene fragments from 11 bacterial strains included according to Heuer *et al*. [38]. Samples are enumerated with the same number in DGGE and the UPGMA tree.

**Fig 8:**
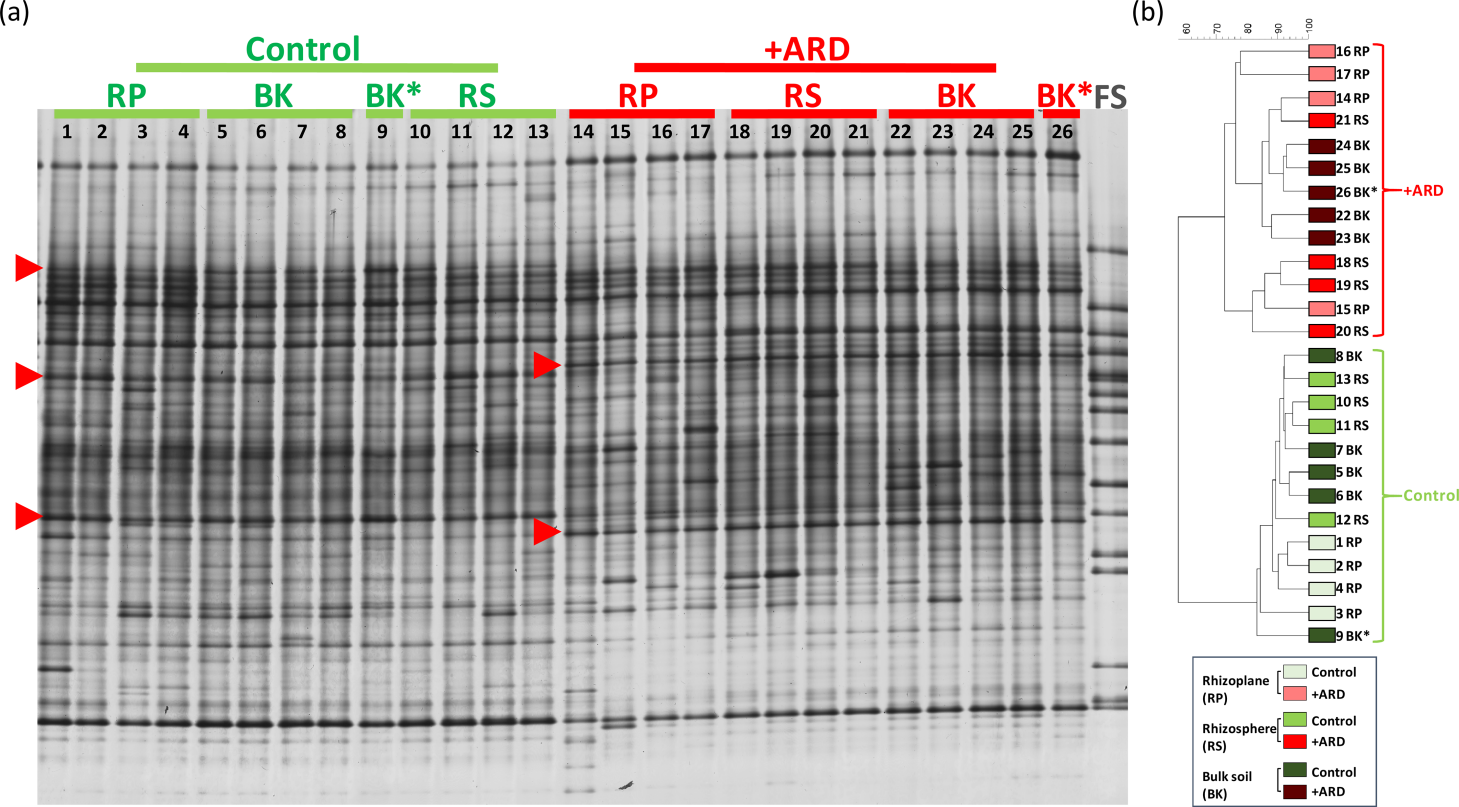
Fungal response to ARD depending on the microhabitat studied. Fungal DGGE fingerprints based on ITS-fragments amplified from total genomic DNA extracted from different microhabitats (Rhizoplane/RP, Rhizosphere/RS and Bulk soil/BK) from plants grown in Control and +ARD soils at the same time. FS: Fungal standard of ITS gene fragments from 16 fungal strains. On the right side the respective UPGMA cluster analysis of the samples is shown. Red arrows indicate fungal populations that are enriched in the respective substrate. Samples are enumerated with the same number in DGGE and UPGMA tree.

**Fig 9:**
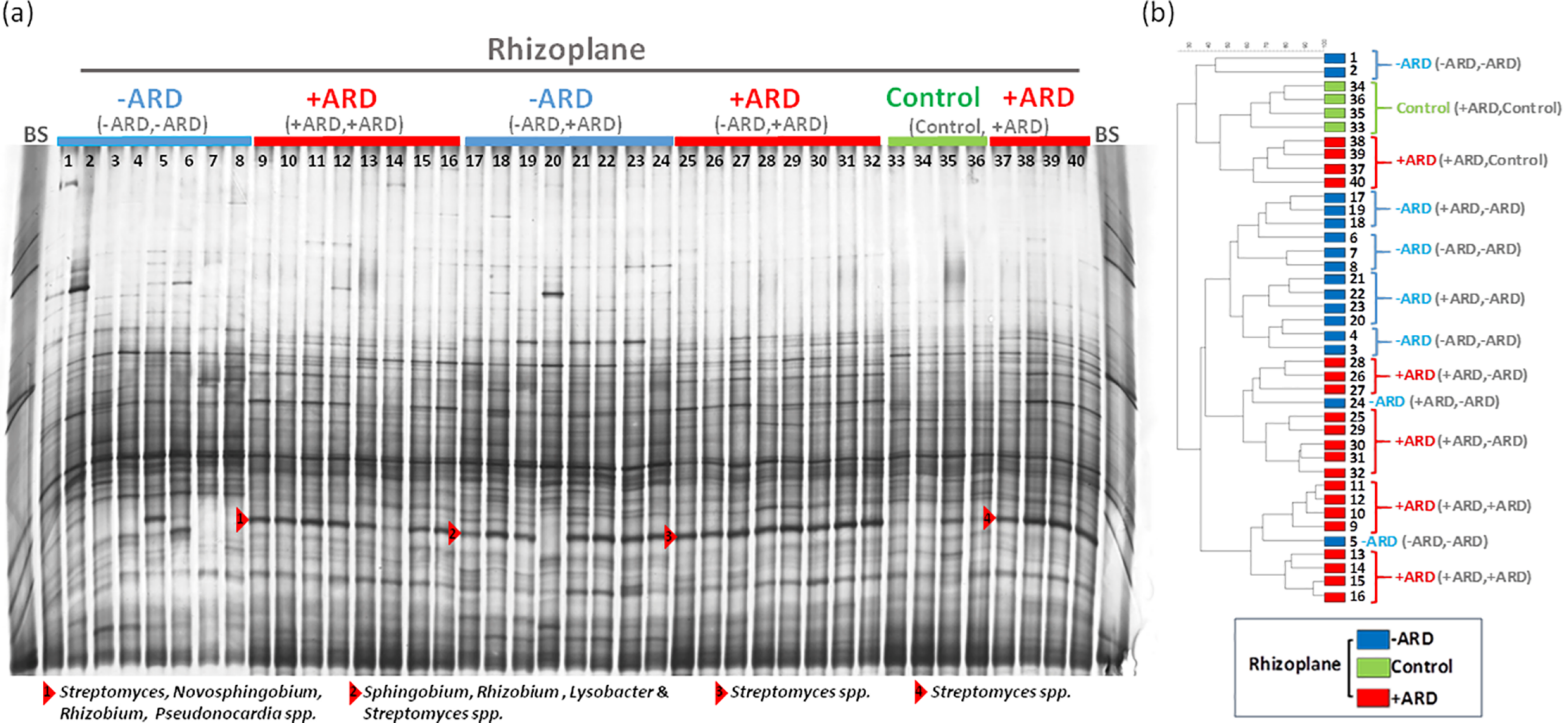
Rhizoplane bacterial response to different substrates. Bacterial DGGE fingerprints based on 16S rRNA gene fragments amplified from total genomic DNA extracted from plants grown in the different substrates (+ARD, -ARD and Control), coming from different combinations of substrates: +ARD, +ARD; -ARD, -ARD; -ARD, +ARD and +ARD, Control. On the right side the respective UPGMA cluster analysis of the samples. Red arrows indicate differentiating bands detected only in the bacterial fingerprint of ARD soils. The bands were excised, reamplified, cloned and sequenced (S2 Fig). BS: Bacterial standard of 16S rRNA gene fragments from 11 bacterial strains included according to Heuer *et al*. [38]. Samples are enumerated with the same number in DGGE and UPGMA tree.

The bacterial community 16S rRNA gene fingerprints displayed a complex banding pattern. They showed, for all treatments, enrichments of some populations in the rhizosphere and in the rhizoplane indicating a lower evenness and richness, in particular in the rhizoplane samples (Fig 7). The comparison of the fingerprints for rhizosphere soil and rhizoplane, respectively, showed no significant differences when both compartments were filled with the same substrate (+ARD/+ARD or -ARD/-ARD) (Table 1). However, significantly different fingerprints were observed between the respective rhizosphere and rhizoplane fingerprints, when the compartments were filled with different substrates (-ARD/+ARD and Control/+ARD). These differences in the microbiome composition were investigated for +ARD/Control (Fig 1) for different taxonomic groups (Fig 7; Table 2). Significant differences were observed between +ARD and Control fingerprints for rhizoplane, rhizosphere and bulk soil, respectively (Fig 7). A marked rhizosphere effect was observed in +ARD with some strong populations and three dominant bands were detected only in the rhizoplane of the +ARD but not in Control samples (see marked bands in Fig 7). In the rhizosphere of roots exposed to +ARD only one band was detected in all bacterial fingerprints that was not detected in Control soil. Interestingly, the differences between bacterial fingerprint of +ARD and Control were lowest in bulk soil, while those differences between the fungal fingerprints were highest in bulk soil (Fig 8; Table 2). In contrast to the bacterial fingerprints no enrichment of particular fungal populations was observed in the rhizoplane or rhizosphere, indicating a less pronounced rhizosphere effect for fungi (Fig 8). Indeed, most of the fungal fingerprints bands were shared between soil microhabitats. In the +ARD samples two distinct bands were detected in all samples which were not detected in the Control (see bands marked in Fig 8). In addition, a higher variability between replicates was observed. UPGMA revealed distinct separate clusters for fungal communities of +ARD and Control soil with rather high similarity of rhizoplane, rhizosphere and bulk soil samples. Fig 9 shows the DGGE fingerprints of rhizoplane samples of -ARD/-ARD, +ARD/+ARD, -ARD (-ARD/+ARD), +ARD (-ARD/+ARD), Control (Control/+ARD) and +ARD (Control/+ARD). Interestingly, one band was detected in -ARD rhizoplane fingerprints only when the second compartment was +ARD. The fingerprints of the -ARD bulk and rhizosphere samples showed higher variability among replicates (data not shown).

**Table 1:** Substrate effect on the microbial community structure in the rhizosphere and rhizoplane. Differences (dissimilarity-values) between the DGGE fingerprints from the substrates within each variant (-ARD, -ARD), (+ARD, +ARD), (+ARD, -ARD) (+ARD, Control) in two microhabitats (Rhizoplane, rhizosphere). D-values were calculated based on pairwise Pearson’s similarity coefficients. Significant differences are indicated by asterisk (p<0.05) and n.s. = not significant difference.

	(-ARD/-ARD)	(+ARD/+ARD)	(+ARD/-ARD)	(+ARD/Control)
Rhizoplane	n.s	n.s	19.17*	19.48*
Rhizosphere	n.s	n.s	17.86*	20.12*

**Table 2:** Substrate effect on different taxonomic groups in rhizoplane, rhizosphere and bulk soil. Differences (dissimilarity-values) between the DGGE fingerprints (Fungi: Fig 8, Bacteria: Fig 7, other DGGEs not shown) from the substrates within the treatment (+ARD/Control). N.d = not determined. D-values were calculated based on pairwise Pearson similarity coefficients. Significant differences are indicated by asterisk (p<0.05).

	Fungi	Bacteria	Actino-bacteria	Beta-proteo-bacteria	Alpha-proteo-bacteria	Pseudo-monas	Bacillus	Strepto-myces
Rhizoplane	21.45*	24.58*	56.21*	34.72*	32.36*	4.88*	12.03*	21.86*
Rhizosphere	26.87*	25.53*	15.53*	27.70*	23.52*	n.d	n.d	15.60*
Bulk soil	31.22*	4.20*	18.24*	14.55*	n.d	n.d	n.d	12.41*

#### Sequence analysis of differentiating DGGE bands

One of our goals was to identify bacterial populations more enriched in the rhizoplane of plants grown in +ARD soils in comparison to Control and/or–ARD soils (-ARD, -ARD; bands 1–5, Figs 7 and 9 marked by red arrow). Therefore, two additional DGGE fingerprints were carried out to excise these bands (S2 and S3 Figs; identical samples as shown in Figs 7 and 9). For all clones (except 5 and 7 derived from band 2), the closest NCBI hits belonged to the same genus but differed at species level. Sequences of clones 5 and 7 showed, in contrast, high similarity with species belonging to two different genera (*Lysobacter* and *Pseudoxanthomonas*) that are phylogenetically closely related. Bands 1 to 4, although having the same electrophoretic mobility, were excised and analyzed separately according to the treatment. A very intense band was detected (Fig 9) in +ARD soils but not in Control and–ARD soils (-ARD, -ARD). Interestingly the same band was as well highly enriched in–ARD soils when the neighboring compartment contained +ARD soil, suggesting a migration of a specific population from the +ARD soil to the–ARD soil. Different clones of this band excised from the different substrates/treatments were affiliated to the genus *Streptomyces* (97–98% identity). For some treatments even all clones were affiliated to *Streptomyces spp*.: +ARD (+ARD, -ARD), +ARD (Control, +ARD) (Table 3). However, in +ARD soils (+ARD, +ARD) additional sequences were affiliated to the genera *Novosphingobium*, *Rhizobium* and *Pseudonocardia*. Within–ARD soils (-ARD, +ARD), besides *Streptomyces* sequences, some clones were affiliated to genera like *Sphingobium* (3 clones from the 7 analysed) and *Lysobacter* (2 clones from the 7 analysed). Although the investigated band was mostly assigned to *Streptomyces*, other populations were assigned to other genera (*Sphingobium* and *Rhizobium Lysobacter* spp.). In conclusion, the sequencing data suggest that different bacterial populations contributed to the very intensive bands indicated in Fig 9.

**Table 3:** Putative phylogenetic affiliation of 16S rRNA partial gene sequences (V6-V8 region) from the rhizoplane of the bands excised from DGGE (Figs 7 and 9).

Figure/ Band^¥^	Treatment	Clones ^ȣ^	Length	F^ç^	Closest phylogenetic relatives ^§^		
					Identity	Accession no.	QC|ID|E-v
F.9/1	+ARD (+ARD,+ARD)	27^A^-[MH488739]	402bp	1/4	*Streptomyces araujoniae* ASBV-1^T^	NR_125527.1	100%|98%|0.0
		14-[MH488748]	395bp	1/4	*Novosphingobium resinovorum* NCIMB 8767^T^	NR_044045.1	100%|99%|0.0
		17-[MH488750]	392bp	1/4	*Rhizobium galegae* NBRC 14965^T^	NR_113713.1	100%|99%|0.0
		16-[MH488746]	393bp	1/4	*Pseudonocardia acaciae* GMKU095^T^	NR_044580.1	100%|98%|0.0
F.9/2	-ARD (+ARD,-ARD)	2-[MH488749]	392bp	1/7	*Rhizobium mesosinicum* CCBAU 25010^T^	NR_043548.1	100%|98%|0.0
		3 ^B^-[MH488747]	395bp	3/7	*Sphingobium scionense* WP01^T^	NR_116123.1	100%|99%|0.0
		4 ^B^-[MH488747]					
		6 ^B^-[MH488747]					
		5^C^-[MH488753]	395bp	2/7	*Lysobacter ginsengisoli Gsoil 357*^*T*^	NR_112563.1	100%|99%|0.0
		7^C^-[MH488753]					
		8-[MH488742]	402bp	1/7	*Streptomyces araujoniae* ASBV-1^T^	NR_125527.1	100%|98%|0.0
F.9/3	+ARD (+ARD,-ARD)	9-[MH488743]	401bp	5/5	*Streptomyces araujoniae* ASBV-1^T^	NR_125527.1	100%|97%|0.0
		10^A^-[MH488739]	402bp				100%|98%|0.0
		11^A^-[MH488739]	402bp				100%|98%|0.0
		12-[MH488740]	402bp				100%|97%|0.0
		13-[MH488741]	402bp				100%|98%|0.0
F.9&F.7/4	+ARD (Control,+ARD)	18-[MH488744]	402bp	2/2	*Streptomyces araujoniae* ASBV-1^T^	NR_125527.1	100%|98%|0.0
		19^A^-[MH488739]	402bp				
F.7/5	+ARD (Control,+ARD)	20^D^-[MH488751]	393bp	3/3	*Variovorax paradoxus* 13-0-1D^T^	NR_036930.1	100%|100%|0.0
		21^D^-[MH488751]					
		22-[MH488752]	392bp		*Variovorax paradoxus* 13-0-1D^T^	NR_036930.1	100%|99%|0.0

¥ Indicates the figure showing the band excised for identification. Each number represents a pool of bands extracted with the same melting behaviour and the same treatment. Each pool of bands has been assigned a different number that goes from 1 till 5.

ȣ Genbank sequence accession numbers of clones given in brackets. Clones with same letters possess exactly the same partial gene sequence.

Ç Number of the cloned sequences matching the same species in comparison to the total number of positive clones obtained from one pool of bands of the same treatment.

§ Identity of strain with the most closely related bacterial sequence; Accession no: Genbank sequence accession number of the closely related bacterial strain. QC|ID|E-v: QC = Query cover; ID = Identity; E-v = E-value.

The band number 5 in Fig 7 that was present in the rhizoplane from plants grown in +ARD soils but not in rhizoplane from Control was identified based on the sequence of all three clones obtained, as *Variovorax paradoxus* (99–100% identity).

## Discussion

The split-root approach enabled us to evaluate substrate-specific effects on root growth and shoot growth of the same plant. In the present experiment plants grown in +ARD/-ARD soil showed shoot growth comparable to plants grown in -ARD/-ARD only, while those from +ARD/+ARD rhizoboxes showed poor shoot growth. Obviously, spatial separation of +ARD and -ARD soil allowed reducing negative effects on shoot growth while experiments using dilution of +ARD soil with sterilized (or soil never planted to apple) at similar or even higher rates (20 to 95% healthy soil) were not successful in overcoming ARD [2,15,18,20,39]. Also for root growth, compensation was observed in -ARD/+ARD or control/+ARD treatment, i.e. if one of the compartments was filled with -ARD soil or control soil, root growth in the +ARD compartment was enhanced compared to +ARD/+ARD. From these results it can be concluded that the response of apple to ARD soil is not systemic as mainly roots in direct contact with +ARD soil were strikingly affected in their growth and morphology as well as in their ability to take up ^15^N. Based on this observation we propose that only direct exposure to the +ARD microbiome and its metabolites, e.g. secreted molecules, volatiles and local plant defence responses, seemed to cause the changes in root morphology. This is in line with field observations as reported by Hoestra [2], showing that ARD mainly affects the apple trees in the first years of planting, thereafter the roots grow deeper into soil layers less affected by ARD. It must be emphasized that this non-systemic response was observed despite strong indications that some bacterial populations (*Streptomyces*, *Sphingobium*) were detected in the -ARD compartment only when the other compartment contained +ARD soil (Fig 9). This finding might either be explained by a migration of respective bacterial populations via the plant or due to their enrichment caused by root metabolites in the -ARD compartment only, when the other part of the apple roots were exposed to +ARD. Exchange between the two compartments of the split-root system was revealed by the increase of ^15^N abundance in the root tissue grown in the unlabelled compartment of treatment -ARD/+ARD. Whatever caused the enrichment of these populations, they themselves are likely not the causal agent of ARD as the roots in the -ARD compartment were not affected.

Studies reported that the rhizosphere microbiomes of apple rootstocks or seedlings were significantly different when seedlings were grown in +ARD soil compared to heat-treated [8] or γ-irradiated soil [40]. However, in these studies different plant individuals were assessed. In the split-root experiments reported here we show that one individual plant being exposed to different substrates showed drastic local changes of the rhizoplane and rhizosphere microbiome. With the exceptions mentioned above, however, only upon direct exposure.

We conclude that the mobility of the ARD-causing agents in soil is low and that it is unlikely to migrate within the root system. This is in line with the conceptual model developed by Emmett *et al*. [4], which investigated the relationship between root order, pathogen DNA abundance and phenolic profiles. They hypothesized that roots in primary development and transitioning to secondary development have the highest pathogen abundance, while plant chemical defences constitutively allocated to higher order roots protect the vascular tissues and hence the spread throughout the root system.

In the split-root approach, shoot growth was only reduced if both parts of the root system were growing in +ARD soil. Root growth declined to a much lesser extent if the second half of the root system was growing in -ARD or Control soil. However, the roots growing in +ARD soil, despite this growth compensation, still showed significantly lower nitrate uptake activity as indicated by the lower ^15^N abundance in root and shoot tissue when the ^15^N label was applied to the +ARD side of the treatment. This decrease of nitrate uptake activity is not a result of decreased nitrogen demand as shoot size was the same for the crosswise labelled treatments. Whether ARD is specifically inhibiting nitrate uptake or root uptake activity in general cannot be concluded from the present data. ^15^N labelled nitrate was chosen in the first place as nitrogen requirement of young seedlings is high, and ^15^N isotopes are stable and relatively easy to measure. Nitrate instead of ammonium was used to avoid any possible confounding effect with alterations of nitrification potential between +ARD and -ARD.

It has been reported that roots growing in ARD soil show brownish discolouration, necrotic cortex and epidermis and only few root hairs [2,8,40]. Similar discolouration was observed in the present experiment. However, as reported by others [2] some root tips were still white and growing. In general, roots extracted from +ARD soil were more brittle, a phenomenon observed during our sampling procedure.

A more mechanistic explanation of the observed changes in root appearance comes from recent, comparative transcriptome analysis of roots from apple 'M26' grown in +ARD and -ARD soil reported by Weiß *et al*. [8]. Massive sequencing of cDNA ends (MACE) and RT-qPCR revealed that roots of apple plants exposed to ARD soil showed an up-regulated expression of genes coding for secondary metabolite production as well as plant defence, regulatory and signalling genes. This is in line with Yim *et al*. [40] proposing that damaged +ARD roots invest more energy in defence reactions. The observations of Weiß *et al*. [8] were similar to those of Shin *et al*. [41]. In their transcriptome studies they showed specific molecular response of apple roots to *Pythium ultimum* and identified genes involved in infection-induced production of pathogenesis-related proteins and several antimicrobial secondary metabolites. Moreover, ethylene, jasmonate and cytokinin signaling was indicated to play a role in the defence response [41].

The dark coloration of ARD roots is easily confounded with the red-brown coloration of apple root segments as they increase in age during ontogeny (Fig 1). High concentrations of phloridzin and quercetin, which may cause the discolouration, are typical of apple roots, and reduced growth rates may indirectly result in increased concentrations. Henfrey *et al*. [42] showed that phenolic compounds accumulated in plants exposed to +ARD. A potential role as an antioxidant substance was proposed by these authors. In addition, a higher level of the flavonoid phloridzin was found in root exudates of +ARD plants [3].

The changes in the root morphology and exudation patterns that occurred in response to the growth in +ARD soil likely shaped the rhizoplane and rhizosphere microbiome. At the same time a so-called soil memory effect [43] due to previous growth of apple might have shaped the soil microbiome and the abundance of likely resting stages of potential pathogens.

The DGGE fingerprints as well as the qPCR data indicated that sterilization by γ-radiation reduced bacterial and fungal diversity and abundances. The higher variability between replicates of DGGE fingerprints of -ARD is most likely due to the lower abundance of target sequences. The -ARD soil was not sterile at the time of sampling. The colonization of the -ARD/-ARD compartments might be due to plant-, air- or irrigation-derived microorganisms. An additional Control soil was used in this split-root experiment. Although the soil was taken from the same site, its microbial communities were likely influenced by grass growth. The importance of the soil with its associated microbiome for plant growth was most impressively observed when root growth in the compartments with Control soil was compared to the +ARD compartment.

The comparison of the DGGE fingerprints clearly revealed a strong enrichment of a few soil populations in the rhizosphere which was far more pronounced in the rhizoplane of apple plantlets. A reduced diversity in the rhizosphere was described for many plant species previously and it is readily accepted that root exudates shape the rhizosphere microbiome [22,44].

DGGE fingerprints appeal as a straightforward tool to compare fingerprints of bacteria and fungi present in the different microhabitats (bulk, rhizosphere and rhizoplane) as well as in the different substrates (+ARD, -ARD, Control). Sequencing of bands from the bacterial DGGE fingerprints (Fig 7 and Fig 9) indicated that diverse bacterial populations contributed to the differentiating band that was strikingly increased in abundance in the +ARD compartment. In all rhizoboxes with +ARD *Streptomyces (Actinomycetales)* were detected as dominant population, contributing to the differentiating band. Actinomycete-like microorganisms were reported previously to be constantly present not only in the root lesions from apple seedlings grown in ARD soils but also inhabiting the margins were the lesion begins [45], being able to invade the cortical layers till the endodermis, suggesting a pathogenic behaviour [45].

In contrast, the less intense but clearly differentiating band was only represented by *Variovorax*. Interestingly, bacteria belonging to *Variovorax* were isolated more frequently from apple grown in ARD in comparison with plants grown in control soils [46]. In conclusion, data suggest that plants show no systemic response to ARD although transport and / or exchange between the two parts occurred as indicated by ^15^N and microbial data. The split-root experiment provided a better understanding of the close links between soil, microbiome and plant.

## Supporting information

S1 Table Primer used in this study for DGGE and qPCR analyses. (DOCX)

S1 Fig Correlation between root lengths derived from scanning the split-root box surface on day 33, and root lengths determined by destructive sampling on day 34. (DOCX)

S2 Fig Bacterial DGGE fingerprints of rhizoplane samples (corresponds to Fig 9) with excised differentiated bands indicated. (TIF)

S3 Fig Bacterial DGGE fingerprints of samples from different microhabitats (corresponds to Fig 7) with excised differentiated bands indicated. (TIF)
