# Supplementary material for: Root growth, function and rhizosphere microbiome analyses show local rather than systemic effects in apple plant response to replant disease soil
Source: PLoS One. 2018 Oct 8;13(10):e0204922. doi: 10.1371/journal.pone.0204922 (PMC6175279; doi:10.1371/journal.pone.0204922)
Supplement: S1 Table — (DOCX) [file pone.0204922.s001.docx]

**S1 Table. Primer used in this study for DGGE and qPCR analyses.**

|  | PCR target | Primer pair | Sequence (5‘-3‘) | Annealing temp. (°C) | Reference |
| --- | --- | --- | --- | --- | --- |
| *DGEE* | ***16S rRNA gene fragment*** | | | | |
|  | *Bacteria* | F984GC*  R1378 | GC clamp* GAACGCGAAGAACCTTAC  CGGTGTGTACAAGGCCCGGGAACG | 53 | Nübel *et al.* [1]  Heuer *et al.* [2] |
|  | *Pseudomonas* | F311Ps  R1459Ps | CTGGTCTGAGAGGATGATCAGT  AATCACTCCGTGGTAACCGT | 63 | Milling *et al.* [3] |
|  | *Alphaproteobacteria* | F203α  R1494 | CCGCATACGCCCTACGGGGGAAAGATTT  CTACGGNTACCTTGTTACGAC | 56 | Gomes *et al.*  [4]  Weisburg *et al.* [5] modified by Heuer *et al.* [6] |
|  | *Betaproteobacteria* | F948β | CGCACAAGCGGTGGATGA | 64 | Gomes *et al.* [4] |
|  | *Actinobacteria* | F243HGC | GGATGAGCCCGCGGCCTA | 63 | Heuer *et al.* [2] |
|  | *Bacillus* | Bac F | GGGAAACCGGGGCTAATACCGGAT | 65 | Garbeva *et al.* [7] |
|  | *Streptomyces* | F126  R1423 | GCCCTGCACTCTGGGACAAGC  GTTAGGCCACCGGCTTCG | 62 | Weinert *et al.* [8] |
|  | ***Fungal ITS fragment*** | | | | |
|  | *Fungi nested* | ITS1F  ITS4 | CTTGGTCATTTAGAGGAAGTAA  TCCTCCGCTTATTGATATGC | 55 | Anderson & Cairney [9] |
|  | *Fungi* | ITS1FGC** | GCclamp** CTTGGTCATTTAGAGGAAGTAA | 55 | Anderson & Cairney [9] |
|  |  | ITS2 | GCTGCGTTCTTCATCGATGC |  |  |
| qPCR | *Bacteria* | Bact1369F  Prok1492R  TM1389F  (5’FAM,3’TAMRA) | CGGTGAATACGTTCYCGG  GGWTACCTTGTTACGACTT  CTTGTACACACCGCCCGTC | 56 | Suzuki *et al.* [10] |
|  | *Fungi* | ITS1  ITS4 | TCCGTAGGTGAACCTGCGG  TCCTCCGCTTATTGATATGC | 50 | Gschwendtner *et al.* [11]; modified according to Vogel *et al.* [12] |
| *GC clamp bacteria: CGC-CCG-GGG-CGC-GCC-CCG-GGC-GGG-GCG-GGG-GCA-CGG-GGG  **GC clamp Fungi: CGC-CCG-CCG-CGC-GCG-GCG-GGC-GGG-GCG-GGG-GCA-CGG-GGG-G | | | | | |

References

1. Nübel U, Engelen B, Felske A, Snaidr J, Wieshuber A, Amann RI, et al. Sequence heterogeneities of genes encoding 16S rRNAs in Paenibacillus polymyxa detected by temperature gradient gel electrophoresis. Journal of Bacteriology. 1996; 178: 5636–5643.

2. Heuer H, Krsek M, Baker P, Smalla K, Wellington EM. Analysis of Actinomycete Communities by Specific Amplification of Genes Encoding 16S rRNA and Gel-Electrophoretic Separation in Denaturing Gradients. Applied and Environmental Microbiology. 1997: 3233–3241.

3. Milling A, Smalla K, Maidl FX, Schloter M, Munch JC. Effects of transgenic potatoes with an altered starch composition on the diversity of soil and rhizosphere bacteria and fungi. Plant Soil. 2005; 266: 23–39. doi: 10.1007/s11104-005-4906-4.

4. Gomes NCM, Heuer H, Schönfeld J, Costa R, Mendonça-Hagler L, Smalla K. Bacterial diversity of the rhizosphere of maize (Zea mays) grown in tropical soil studied by temperature gradient gel electrophoresis. Plant Soil. 2001: 167–180.

5. Weisburg WG, Barns SM, Pelletier DA, Lane DJ. 16S ribosomal DNA amplification for phylogenetic study. Journal of Bacteriology. 1991; 173: 697–703.

6. Heuer H, Kroppenstedt RM, Lottmann J, Berg G, Smalla K. Effects of T4 Lysozyme Release from Transgenic Potato Roots on Bacterial Rhizosphere Communities Are Negligible Relative to Natural Factors. Applied and Environmental Microbiology. 2002; 68: 1325–1335. doi: 10.1128/AEM.68.3.1325-1335.2002.

7. Garbeva P, van Veen JA, van Elsas JD. Predominant Bacillus spp. in Agricultural Soil under Different Management Regimes Detected via PCR-DGGE. Microbial Ecology. 2003; 45: 302–316. doi: 10.1007/s00248-002-2034-8.

8. Weinert N, Meincke R, Gottwald C, Heuer H, Gomes NCM, Schloter M, et al. Rhizosphere Communities of Genetically Modified Zeaxanthin-Accumulating Potato Plants and Their Parent Cultivar Differ Less than Those of Different Potato Cultivars. Applied and Environmental Microbiology. 2009; 75: 3859–3865. doi: 10.1128/AEM.00414-09.

9. Anderson IC, Cairney JWG. Diversity and ecology of soil fungal communities: increased understanding through the application of molecular techniques. Environ Microbiol. 2004; 6: 769–779. doi: 10.1111/j.1462-2920.2004.00675.x.

10. Suzuki MT, Taylor LT, DeLong EF. Quantitative Analysis of Small-Subunit rRNA Genes in Mixed Microbial Populations via 5′-Nuclease Assays. Applied and Environmental Microbiology. 2000; 66: 4605. doi: 10.1128/AEM.66.11.4605-4614.2000.

11. Gschwendtner S, Reichmann M, Müller M, Radl V, Munch JC, Schloter M. Effects of genetically modified amylopectin-accumulating potato plants on the abundance of beneficial and pathogenic microorganisms in the rhizosphere. Plant Soil. 2010; 335: 413–422. doi: 10.1007/s11104-010-0430-2.

12. Vogel C, Babin D, Pronk GJ, Heister K, Smalla K, Kögel-Knabner I. Establishment of macro-aggregates and organic matter turnover by microbial communities in long-term incubated artificial soils. Soil Biology and Biochemistry. 2014; 79: 57–67. doi: 10.1016/j.soilbio.2014.07.012.
