# Supplementary material for: Root growth, function and rhizosphere microbiome analyses show local rather than systemic effects in apple plant response to replant disease soil
Source: PLoS One. 2018 Oct 8;13(10):e0204922. doi: 10.1371/journal.pone.0204922 (PMC6175279; doi:10.1371/journal.pone.0204922)
Supplement: S1 Fig — (DOCX) [file pone.0204922.s002.docx]

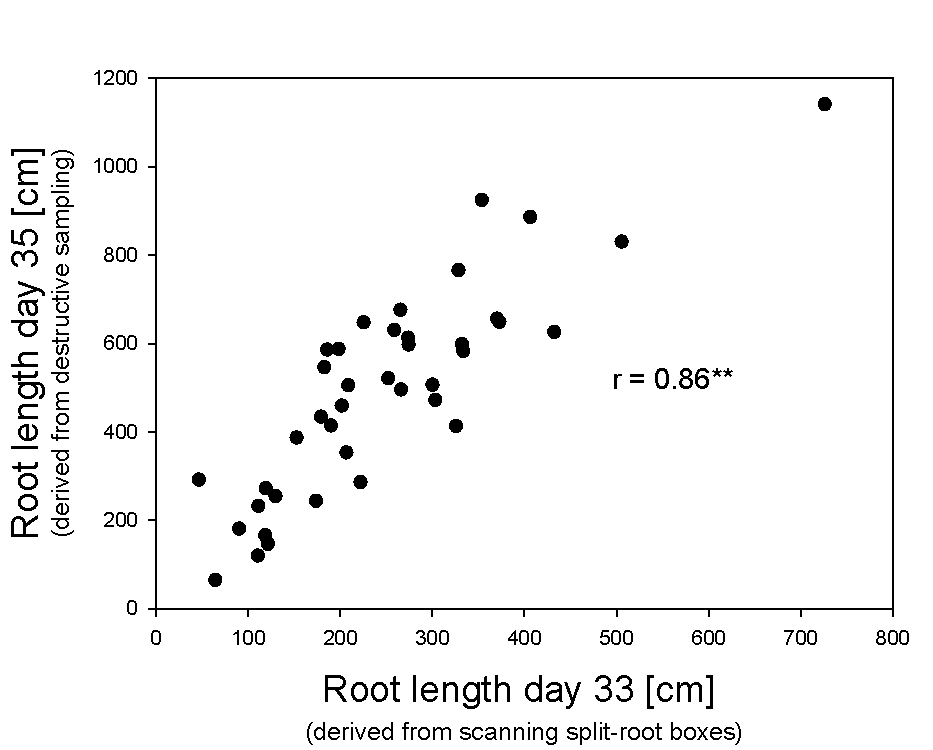


**S1 Fig. Correlation between root lengths derived from scanning the split-root box surface on day 33, and root lengths determined by destructive sampling on day 34**. Pearson correlation coefficient is based on n=20.
