# Supplementary figures and images for: Root growth, function and rhizosphere microbiome analyses show local rather than systemic effects in apple plant response to replant disease soil
Source: PLoS One. 2018 Oct 8;13(10):e0204922. doi: 10.1371/journal.pone.0204922 (PMC6175279; doi:10.1371/journal.pone.0204922)

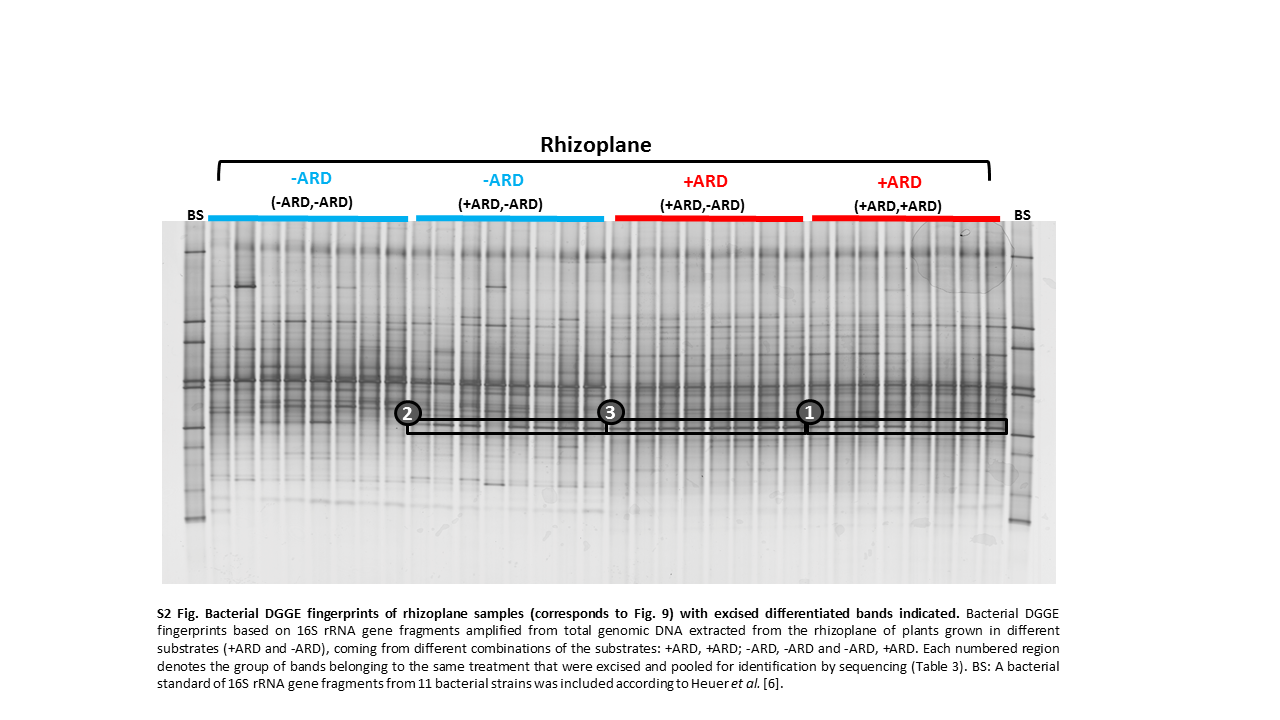

Supplement: S2 Fig — (TIF) [file pone.0204922.s003.tif]

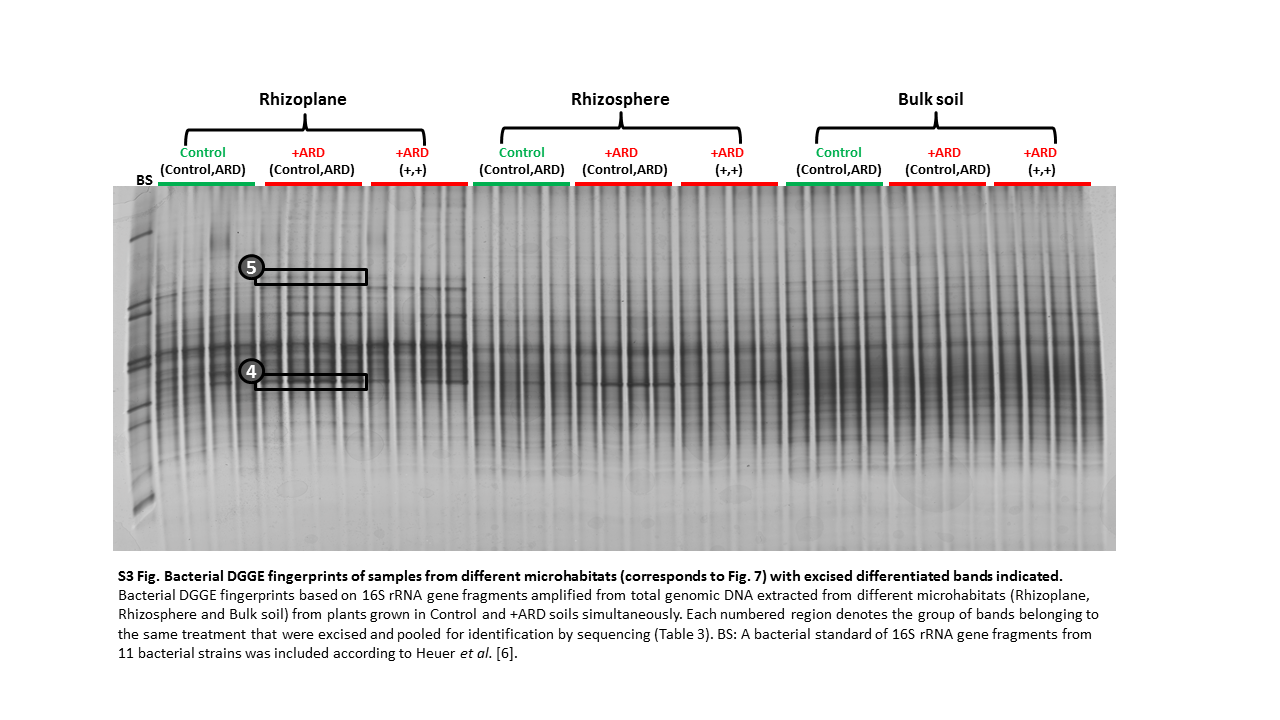

Supplement: S3 Fig — (TIF) [file pone.0204922.s004.tif]
